# Immunoregulatory mechanisms in parasitic eosinophilic lung disease: the role of IgE immune complexes and NLRC4 inflammasome

**DOI:** 10.3389/fimmu.2026.1776407

**Published:** 2026-08-26

**Authors:** Yuanchao Cao, Dingyu Rao, Zongbo Peng, Zihao Chen, Qinghui Xia, Chunfa Xie, Yanglin Fu, Defa Huang, Zuxiong Zhang, Chuan Yao

**Affiliations:** 1 Department of Cardiothoracic Surgery, The Affiliated Hospital of Jiujiang University, Jiujiang, China; 2 Department of Thoracic Surgery, First Affiliated Hospital of Gannan Medical University, Ganzhou, China; 3 The First Clinical College, Gannan Medical University, Ganzhou, China; 4 Laboratory Medicine, The First Affiliated Hospital of Gannan Medical University, Ganzhou, China

**Keywords:** eosinophils, FcϵRII, hypothetical immunoregulatory axis, IgE immune complexes, immunoregulation, inflammatory response, NLRC4 inflammasome, parasitic eosinophilic lung disease

## Abstract

Parasitic eosinophilic lung disease is characterized by eosinophil infiltration and dysregulated immune responses, posing significant challenges due to its complex immunoregulatory mechanisms which remain incompletely understood. This review explores the emerging role of IgE immune complexes (IgE ICs) in modulating eosinophilic immune responses, proposing a hypothetical signaling axis involving the NLRC4 inflammasome that may influence disease progression. We synthesize preliminary evidence from cellular models and discuss the potential implications of this pathway for parasitic eosinophilic lung disease. Integrating recent findings, we explore how IgE ICs interact with Toll-like receptor 2 (TLR2) and Fc epsilon receptor II (FcϵRII) to regulate eosinophil inflammatory responses, degranulation, and the expression of associated proteins. These interactions reveal a dual regulatory function of immune complexes in parasitic infections and related pulmonary disorders. By critically examining the available evidence for crosstalk between IgE ICs and the NLRC4 inflammasome, this review aims to provide a conceptual framework and hypothesis-generating perspectives for developing targeted immunotherapeutic strategies for parasitic eosinophilic lung disease. Importantly, we acknowledge that the mechanistic evidence for this axis is primarily derived from *in vitro* studies and requires validation in physiologically relevant systems.

## 1 Introduction

Parasitic infections represent a significant etiological factor in the development of eosinophilic lung diseases, a heterogeneous group of pulmonary disorders characterized by an abnormal accumulation of eosinophils within lung tissues and alveolar spaces. Eosinophilic lung diseases encompass a broad spectrum, including idiopathic forms such as acute and chronic eosinophilic pneumonia, eosinophilic granulomatosis with polyangiitis, and hypereosinophilic syndromes, as well as secondary forms associated with known causes like allergic bronchopulmonary aspergillosis, drug reactions, and parasitic infections (1, 2). Among these, parasitic infections induce a particularly complex immunopathological process due to the migratory lifecycle of helminth larvae through pulmonary tissues, which triggers robust innate and adaptive immune responses.

Eosinophils, bone marrow-derived granulocytes, play a pivotal role in the host defense against parasitic infections and are central to the pathogenesis of eosinophilic lung diseases. Upon parasitic invasion, eosinophils are recruited to the lungs where they recognize pathogen-associated molecular patterns (PAMPs) via pattern recognition receptors (PRRs), including Toll-like receptors (TLRs) and NOD-like receptors (NLRs). This recognition prompts eosinophils to release an array of cytotoxic granule proteins, cytokines, chemokines, and lipid mediators, orchestrating an immune response aimed at parasite clearance but also contributing to tissue inflammation and remodeling (3, 4). The pulmonary phase of helminth infections, such as those caused by Ascaris suum or Strongyloides venezuelensis, involves eosinophilic infiltration that not only mediates parasite killing but also participates in tissue repair processes. However, excessive or dysregulated eosinophilic responses can lead to pathological consequences including fibrosis and lung dysfunction, highlighting the dual role of eosinophils in host defense and immunopathology (3, 5).

Immunoglobulin E (IgE) antibodies are critical mediators in the immune response against helminths and are intimately involved in the regulation of eosinophil functions. IgE forms immune complexes (ICs) with parasitic antigens, which can bind to Fc epsilon receptors (FcϵRs) expressed on eosinophils, modulating their activation state and effector functions. While IgE-mediated activation is well recognized in allergic diseases, its precise regulatory mechanisms in parasitic eosinophilic lung diseases remain incompletely understood. Recent studies have demonstrated that IgE immune complexes can influence eosinophilic responses by engaging intracellular signaling pathways and inflammasome components, thereby modulating the balance between protective immunity and immunopathology (6). This regulation is crucial, as it may determine the extent of eosinophil-mediated tissue damage versus effective parasite clearance.

Among the intracellular pathways implicated in eosinophil regulation, the NLRC4 inflammasome, a member of the NLR family, has emerged as a significant player in controlling eosinophilic inflammation. Inflammasomes are cytosolic multiprotein complexes that detect microbial components and danger signals, leading to the activation of caspase-1 and subsequent maturation and secretion of pro-inflammatory cytokines such as interleukin-1β (IL-1β). NLRC4 inflammasome activation has been shown to occur in eosinophils in response to specific ligands, including bacterial flagellin, and to modulate eosinophil degranulation, cytokine production, and immune regulatory receptor expression (7). The interplay between IgE immune complexes and NLRC4 inflammasome activation suggests a sophisticated mechanism by which eosinophil functions are fine-tuned during parasitic infections, balancing inflammation and tissue homeostasis.

While the NLRP3 inflammasome has been more extensively studied in pulmonary inflammation and type 2 immunity, emerging evidence suggests that NLRC4 may play a distinct, non-redundant role in eosinophil biology. The rationale for focusing on NLRC4 in this review is threefold: (1) NLRC4 is activated by cytosolic bacterial components, which may be relevant during parasitic infections where bacterial translocation or co-infection occurs; (2) unlike NLRP3, NLRC4 is constitutively expressed in resting eosinophils and can be rapidly mobilized; and (3) recent *in vitro* studies have specifically linked NLRC4 to IgE-mediated eosinophil degranulation pathways. It should be emphasized, however, that NLRP3, IL-33 signaling, epithelial alarmins, and type 2 innate lymphoid cell pathways remain considerably more established in eosinophilic pulmonary inflammation, and the proposed NLRC4-centric framework is presented as a complementary hypothesis rather than a replacement for these well-characterized pathways.

Given the complexity of eosinophil-mediated immune responses in parasitic eosinophilic lung diseases, understanding the crosstalk between IgE immune complexes and NLRC4 inflammasome activation is of paramount importance. This regulatory axis may not only elucidate the pathogenesis of eosinophilic lung diseases but also reveal novel therapeutic targets. For instance, modulation of NLRC4 inflammasome activity or interference with IgE immune complex signaling could potentially attenuate eosinophil-driven lung inflammation without compromising host defense. Furthermore, insights into this mechanism may have broader implications for other eosinophil-associated conditions such as allergic asthma and chronic eosinophilic pneumonia.

In this review, we critically examine recent advances in the understanding of the immunoregulatory mechanisms underlying parasitic eosinophilic lung diseases, with particular focus on a proposed role for IgE immune complexes and the NLRC4 inflammasome. Importantly, we acknowledge that much of the mechanistic evidence for this axis derives from *in vitro* studies using eosinophilic cell lines, and its *in vivo* relevance in parasitic disease remains to be established. We explore the cellular and molecular pathways involved, integrating findings from experimental models and clinical observations, while explicitly distinguishing established knowledge from speculative hypotheses.

## 2 Main body

### 2.1 Immunopathological mechanisms of parasitic eosinophilic pneumonia

#### 2.1.1 Biological functions and pathological roles of eosinophils

Eosinophils are multifunctional granulocytes originating from hematopoietic stem cells in the bone marrow and widely distributed in various tissues, particularly at mucosal surfaces such as the gastrointestinal tract, lungs, and skin. Their development and survival are primarily regulated by interleukin-5 (IL-5), which promotes eosinophilopoiesis and activation, as well as by other type 2 cytokines including IL-4 and IL-13. In the context of parasitic infections, eosinophils serve as critical effector cells in host defense, especially against helminths. They accumulate at sites of infection and contribute to parasite clearance through the release of cytotoxic granule proteins such as eosinophil cationic protein (ECP), eosinophil peroxidase (EPO), and major basic protein (MBP). These granule proteins exert direct cytotoxic effects on parasites by damaging their membranes and inducing oxidative stress. Moreover, eosinophils can form extracellular traps composed of DNA and granule proteins, which immobilize and kill pathogens. Beyond direct cytotoxicity, eosinophils modulate immune responses by producing cytokines and chemokines that influence the recruitment and activation of other immune cells, thus orchestrating type 2 immunity.

Eosinophils also exhibit heterogeneity, with distinct subpopulations identified in both steady-state and pathological conditions. For example, resident eosinophils maintain tissue homeostasis and regulate metabolic functions, while inducible eosinophils are recruited during inflammation and exhibit enhanced cytotoxic and pro-inflammatory activities. This plasticity allows eosinophils to participate not only in host defense but also in tissue remodeling and repair processes. In parasitic infections such as filariasis and helminthiasis, eosinophils contribute to controlling parasite burden but may also drive pathology through excessive inflammation and tissue damage, exemplified by conditions like tropical pulmonary eosinophilia.

Pathologically, eosinophils are implicated in a range of eosinophilic disorders characterized by tissue infiltration and inflammation, including eosinophilic asthma, eosinophilic esophagitis, eosinophilic granulomatosis with polyangiitis, and eosinophilic chronic rhinosinusitis. In these diseases, eosinophils release cytotoxic granule proteins and inflammatory mediators that cause tissue injury, remodeling, and fibrosis. For instance, in eosinophilic asthma, activated eosinophils contribute to airway hyperresponsiveness, mucus hypersecretion, and airway remodeling through the secretion of mediators such as ECP and TGF-β. Similarly, in eosinophilic esophagitis, eosinophil-derived proteins disrupt epithelial barrier integrity and promote fibroblast activation, leading to esophageal fibrosis and dysfunction.

Recent advances have highlighted the complex immunoregulatory roles of eosinophils, including their ability to suppress or promote inflammation depending on the microenvironment and disease context. Eosinophils interact with other immune cells such as basophils, T cells, and innate lymphoid cells, influencing the balance between pro-inflammatory and regulatory responses. Moreover, eosinophils express various receptors including CCR3, IL-5 receptor, and pattern recognition receptors like MINCLE, which modulate their recruitment, activation, and effector functions during parasitic infections and allergic inflammation.

Therapeutically, targeting eosinophils has become a cornerstone in managing eosinophilic diseases. Biologics directed against IL-5 or its receptor (e.g., mepolizumab, reslizumab, benralizumab) effectively reduce eosinophil counts and improve clinical outcomes in severe eosinophilic asthma and related disorders. However, it is important to note that these studies were conducted in allergic asthma patients, and the efficacy of these agents in parasitic eosinophilic lung disease has not been established. Furthermore, concerns remain regarding the long-term safety of eosinophil depletion, given their roles in tissue homeostasis and immune surveillance. The heterogeneity of eosinophil subpopulations and their diverse roles in homeostasis and immunity necessitate careful consideration of potential side effects and long-term consequences of eosinophil depletion.

In summary, eosinophils are vital players in host defense against parasitic infections through their cytotoxic and immunomodulatory functions. Their pathological roles in eosinophilic diseases stem from their capacity to induce tissue inflammation and remodeling via granule protein release and cytokine production. Understanding the balance between their protective and detrimental activities is essential for developing targeted therapies that mitigate eosinophil-driven pathology while preserving their beneficial functions (8–18).

#### 2.1.2 Immune environment Th2 response and role of IgE

The T helper type 2 (Th2) immune response plays a central role in orchestrating immunity against parasitic infections, particularly helminths, and is also implicated in allergic diseases characterized by IgE-mediated hypersensitivity. Th2 cells, differentiated from naïve CD4+ T cells under the influence of interleukin-4 (IL-4), produce hallmark cytokines including IL-4, IL-5, and IL-13, which collectively promote B cell class switching to IgE, eosinophil recruitment, and mucus production. The generation of antigen-specific IgE by plasma cells is a critical feature of this response, enabling specific recognition of parasitic antigens and allergens. IL-4 secreted by Th2 cells and follicular helper T (Tfh) cells drives immunoglobulin class switching in germinal centers, with recent evidence highlighting the dominant role of IL-4-producing Tfh cells in controlling IgE responses during humoral immunity (19). The IgE antibodies bind with high affinity to FcϵRI receptors on innate effector cells such as mast cells, basophils, and eosinophils, sensitizing them to subsequent antigen exposure and facilitating rapid effector responses.

IgE not only serves as a recognition molecule but also modulates the function of innate immune cells. Upon allergen crosslinking, IgE-bound mast cells and basophils release preformed mediators and newly synthesized cytokines that contribute to inflammation and tissue remodeling (20). Eosinophils, recruited and activated by Th2 cytokines and IgE complexes, further amplify tissue inflammation and contribute to parasite clearance or allergic pathology. Basophils have been identified as a significant early source of IL-4, which can promote Th2 polarization and IgE production, as demonstrated in allergic horses where basophil-derived IL-4 was elevated upon allergen stimulation (21). This suggests a feedback loop whereby IgE-mediated activation of basophils enhances Th2 responses. Additionally, IgE immune complexes can engage Fc receptors on innate immune cells, modulating their activation and cytokine production, thus shaping the immune milieu.

The Th2/IgE axis is tightly regulated, with various factors influencing its magnitude and duration. For instance, regulatory mechanisms involving dendritic cells (DCs) can modulate Th2 polarization; stimulation of Dectin-1 on myeloid DCs suppresses Th2 responses by altering STAT3, STAT6, and NF-κB signaling pathways, thereby reducing expression of OX40L and Th2 chemokines (22). Furthermore, the balance between Th1 and Th2 responses is critical; interventions that enhance Th1 cytokines such as IL-12 and IFN-γ can suppress Th2-driven IgE production and eosinophilia, as shown in murine asthma models (23, 24). The Th2 response is also influenced by transcription factors such as GATA3 and Blimp-1, which regulate Th2 differentiation and cytokine expression; disruption of these factors can attenuate allergic inflammation (25, 26).

**Figure 1:**
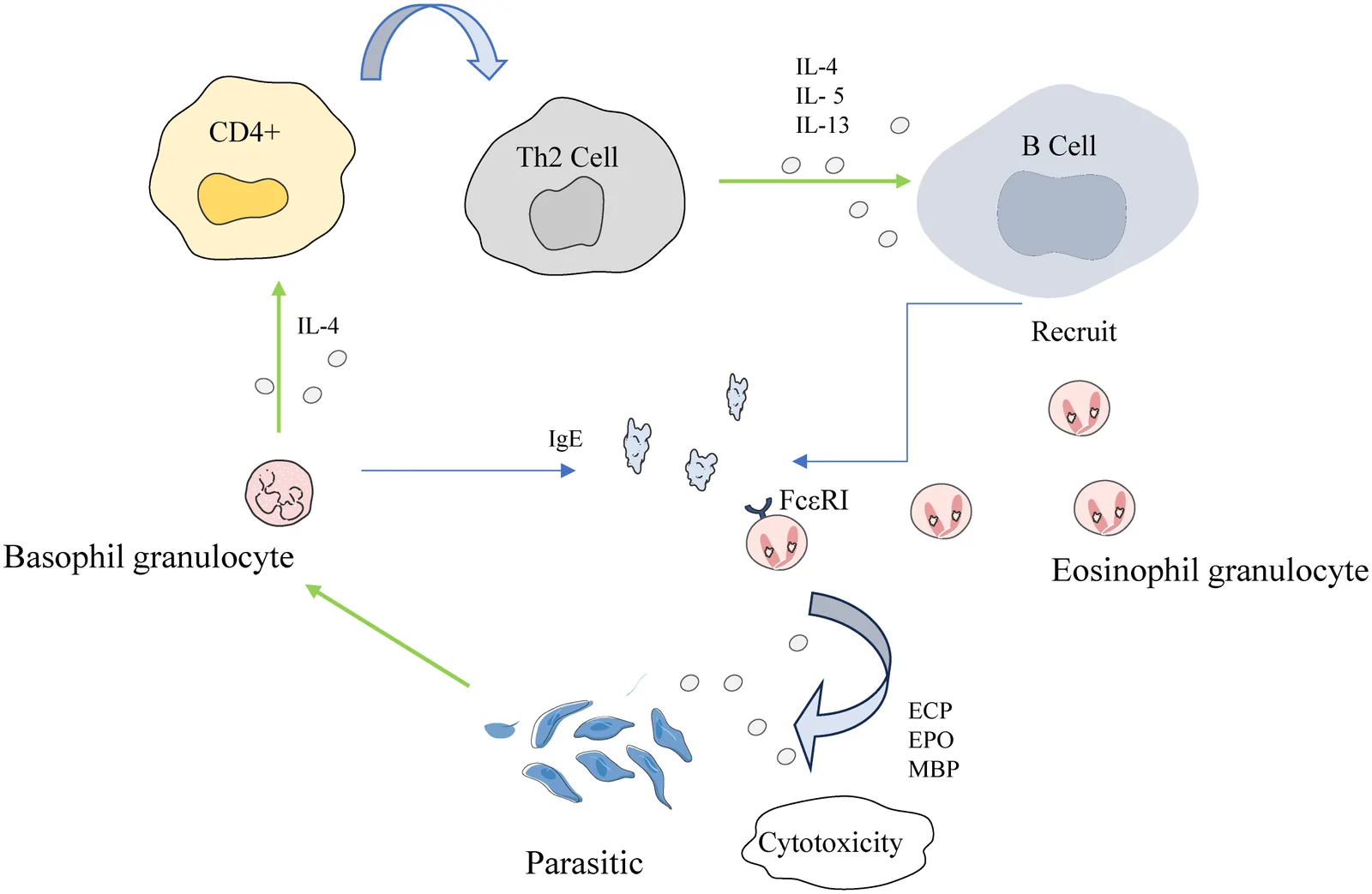
Th2 cell-mediated immune response in parasitic eosinophilic lung disease. Upon parasitic infection, activated CD4+ T helper 2 (Th2) cells secrete interleukin (IL)-4, IL-5, and IL-13. IL-4 drives B cell class switching to produce parasite-specific immunoglobulin E (IgE). IgE binds to the high-affinity receptor (FcϵRI) on basophils and eosinophils, sensitizing these cells. IL-5 and IL-13 are pivotal for eosinophil recruitment, maturation, and activation. Recruited eosinophils exert direct cytotoxicity against parasites by releasing granule proteins, including eosinophil cationic protein (ECP), eosinophil peroxidase (EPO), and major basic protein (MBP).

In the context of parasitic infections, genetic factors modulate the Th2 response and IgE production. Variants in genes such as WSB1 and IL21R have been associated with altered Th2 cytokine levels and parasite-specific IgE responses in children infected with Ascaris lumbricoides, highlighting the genetic basis of immune variability (27). Moreover, nematode parasites secrete immunomodulatory molecules that can skew host immunity towards or away from Th2 dominance, affecting IgE-mediated responses (28) (As shown in **Figure 1**).

Collectively, the Th2 immune environment and IgE antibodies form a tightly interconnected network that enables specific recognition and effector functions against parasites while also contributing to allergic diseases. Understanding the cellular and molecular mechanisms governing this axis, including the role of IgE in activating innate immune cells and modulating their functions, is essential for developing targeted therapies for parasitic infections and IgE-mediated disorders.

#### 2.1.3 Formation of immune complexes and their impact on immune responses

**Figure 2:**
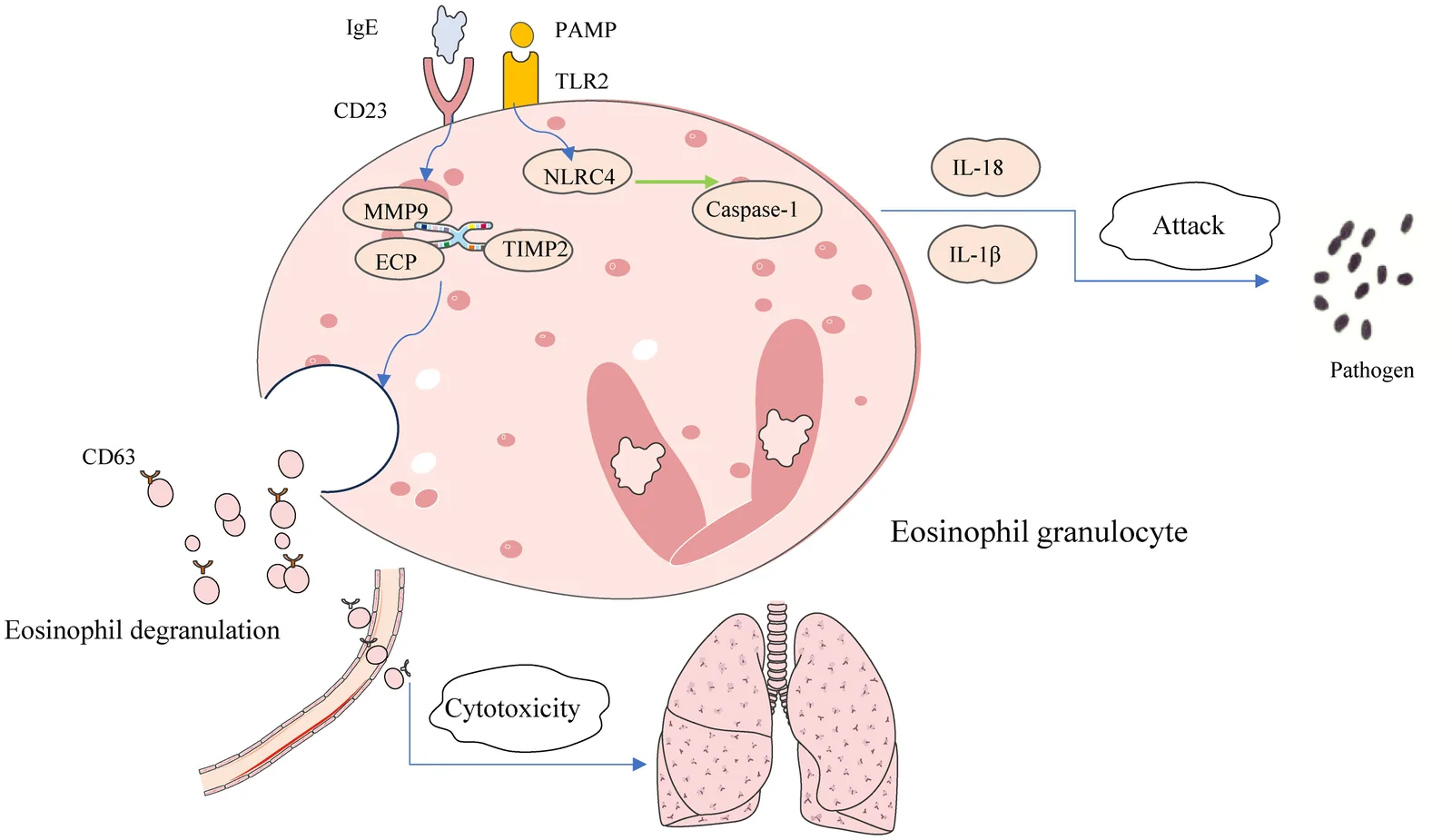
Regulatory of IgE immune complexes and the NLRC4 inflammasome in eosinophils. IgE immune complexes (ICs) bind to the low-affinity IgE receptor FcϵRII (CD23) and synergize with TLR2 signaling within eosinophils. This co-stimulation upregulates the expression of matrix metalloproteinase 9 (MMP9), tissue inhibitor of metalloproteinase 2 (TIMP2), and eosinophil cationic protein (ECP), which are involved in tissue remodeling and cytotoxicity. Concurrently, it induces the expression of NLRC4 inflammasome components. However, IgE ICs also exert a suppressive feedback on fully activated NLRC4 inflammasome assembly, thereby attenuating caspase-1-mediated maturation and secretion of pro-inflammatory cytokines interleukin-1β (IL-1β) and IL-18. This dual regulation fine-tunes eosinophil degranulation and inflammatory output, balancing pathogen clearance with the prevention of excessive tissue damage.

Immune complexes (ICs) are formed when antibodies bind to their specific antigens, creating antigen-antibody complexes that play pivotal roles in modulating immune responses. The formation mechanism typically involves the recognition of antigenic epitopes by the variable regions of immunoglobulins, resulting in multivalent binding and cross-linking that can vary in size and composition depending on antigen and antibody concentrations, affinity, and valency. These complexes have a dualistic function in immune regulation, capable of skewing immune responses toward either pro-inflammatory or anti-inflammatory directions depending on the context of stimulation and the nature of the immune complex. For instance, IgG immune complexes have been extensively studied for their ability to engage Fc gamma receptors (FcγRs) on various immune cells, leading to activation or inhibition of immune responses. Similarly, IgE immune complexes (IgE-ICs) have been implicated in modulating type 2 immune responses, particularly in allergic inflammation and parasitic infections. Recent studies demonstrate that antigen-specific IgE-ICs can induce or suppress immune cell activation through interactions with Fc receptors and pattern recognition receptors (PRRs), such as Toll-like receptors (TLRs) and nucleotide-binding oligomerization domain (NOD)-like receptors (NLRs). The bidirectional effects of ICs are mediated through complex signaling pathways that influence cytokine production, cell degranulation, and gene expression profiles. For example, the engagement of IgE-ICs with FcϵRII (CD23) on B cells can regulate IgE serum levels and antigen presentation, thus modulating adaptive immunity. Moreover, the formation of immune complexes can lead to the activation of inflammasomes, such as the NLRC4 inflammasome in eosinophils, which modulates the release of pro-inflammatory cytokines like interleukin-1β (IL-1β), thereby influencing the inflammatory milieu. The dynamic balance between immune activation and suppression mediated by antigen-antibody complexes underscores their critical role in maintaining immune homeostasis and shaping disease outcomes in contexts such as allergy, autoimmunity, and infection (6, 29, 30) (The complete regulatory network is depicted in **Figure 2**. This schematic illustrates the sequential events from IgE IC binding to downstream effector responses, with the proposed suppressive feedback loop on NLRC4 inflammasome activation indicated in red. Key uncertainties in this model—particularly whether the suppressive effect operates at the level of inflammasome assembly, caspase-1 activation, or downstream cytokine secretion—are highlighted with question marks in the figure and represent priorities for future investigation).

IgE immune complexes exert nuanced effects on eosinophilic responses, serving both activating and inhibitory roles that are crucial in the pathogenesis and resolution of eosinophil-associated diseases. Eosinophils, as granulocytes involved in allergic inflammation and parasitic defense, express Fc receptors including the low-affinity IgE receptor FcϵRII (CD23), which mediates the binding and internalization of IgE-ICs. Studies using the human eosinophilic cell line EoL-1 have revealed that ovalbumin (Ova)-IgE immune complexes can induce the expression of NLRC4 inflammasome components, including NLRC4 itself, caspase-1, and intracellular IL-1β, reportedly leading to the secretion of mature IL-1β via TLR2 signaling pathways. This activation is accompanied by the upregulation of granule proteins such as matrix metalloproteinase 9 (MMP9) and tissue inhibitors of metalloproteinases (TIMP1 and TIMP2), which contribute to tissue remodeling and inflammatory responses. Notably, the presence of Ova-IgE ICs has been reported to suppress NLRC4 inflammasome activation, suggesting an inhibitory feedback mechanism that may temper excessive inflammation. Additionally, co-stimulation with TLR2 ligands and IgE-ICs enhances eosinophil degranulation, as evidenced by increased CD63 expression, a marker of degranulation. These findings suggest that IgE-ICs may modulate eosinophil activity through a delicate balance of activating inflammasome pathways and restraining overactivation, thereby potentially influencing the progression of chronic eosinophilic conditions such as eosinophilic pneumonia, eosinophilic esophagitis, and asthma. However, these observations are derived from a simplified cellular system and require validation in primary eosinophils and *in vivo* models of parasitic infection (6, 30, 31).

### 2.2 Regulatory mechanisms of IgE immune complexes on eosinophils

#### 2.2.1 FcϵRII receptor-mediated signal transduction

FcϵRII, also known as CD23, is a low-affinity receptor for immunoglobulin E (IgE) that is expressed on various immune cells, including eosinophils. The expression of FcϵRII on eosinophils is particularly relevant in the context of parasitic infections and allergic diseases characterized by eosinophilic inflammation. FcϵRII differs from the high-affinity receptor FcϵRI in its binding affinity and functional roles; while FcϵRI primarily mediates immediate hypersensitivity reactions through mast cells and basophils, FcϵRII is implicated in the regulation of IgE serum levels, antigen presentation, and modulation of immune responses. Recent studies have demonstrated that IgE immune complexes (IgE-ICs) preferentially bind to FcϵRII rather than FcϵRI, suggesting a distinct pathway through which IgE-ICs influence immune cell function (30, 32). The binding of IgE-ICs to FcϵRII on eosinophils triggers intracellular signaling cascades that regulate gene expression and protein production critical for eosinophil function and tissue remodeling.

Specifically, FcϵRII engagement by IgE-ICs modulates the expression of matrix metalloproteinase 9 (MMP9), tissue inhibitor of metalloproteinases 2 (TIMP2), and eosinophil cationic protein (ECP), which are key mediators in extracellular matrix remodeling and eosinophil-mediated cytotoxicity. MMP9 is a protease involved in the degradation of extracellular matrix components, facilitating tissue remodeling and inflammatory cell migration. TIMP2 acts as a natural inhibitor of MMPs, maintaining a balance in matrix turnover. ECP is a granule protein released by activated eosinophils that contributes to host defense and tissue damage. The regulation of these molecules by FcϵRII-mediated signaling suggests that IgE-ICs can fine-tune eosinophil activity and influence the pathophysiology of eosinophilic lung diseases, including parasitic eosinophilic pneumonia (33, 34) (As shown in **Figure 2**).

Mechanistically, the cross-linking of FcϵRII by IgE-ICs initiates downstream signaling pathways involving tyrosine kinases and adaptor proteins that lead to transcriptional changes. These changes result in altered mRNA and protein levels of MMP9, TIMP2, and ECP, thereby modulating eosinophil degranulation, migration, and survival. Furthermore, FcϵRII engagement may influence the production of cytokines and chemokines that recruit additional inflammatory cells to the lung tissue, exacerbating eosinophilic inflammation. The dynamic regulation of FcϵRII expression itself is subject to modulation by inflammatory mediators and metalloproteinases such as ADAM10, which can cleave membrane-bound CD23, releasing soluble CD23 (sCD23) that also participates in immune regulation (34, 35).

In addition to its role in eosinophils, FcϵRII on B cells and other antigen-presenting cells facilitates IgE-facilitated antigen presentation, enhancing T cell activation and promoting immune responses. This dual role of FcϵRII in both effector and regulatory functions underscores its importance in allergic and parasitic diseases. Notably, altered FcϵRII expression or function has been implicated in disease states such as chronic spontaneous urticaria and IgG4-related diseases, where dysregulated IgE receptor signaling contributes to pathogenesis (36, 37).

In summary, FcϵRII expressed on eosinophils mediates critical signal transduction pathways upon binding IgE immune complexes, regulating the expression of MMP9, TIMP2, and ECP at both gene and protein levels. This regulation influences eosinophil function, tissue remodeling, and inflammatory responses in parasitic eosinophilic lung diseases. Understanding the molecular details of FcϵRII-mediated signaling offers potential therapeutic targets to modulate eosinophilic inflammation and improve outcomes in parasitic and allergic pulmonary disorders.

#### 2.2.2 Activation and synergistic effects of TLR2 signaling pathway

Toll-like receptor 2 (TLR2) is a critical pattern recognition receptor (PRR) expressed on various immune cells, including eosinophils, where it plays a pivotal role in detecting pathogen-associated molecular patterns (PAMPs) and initiating innate immune responses. Eosinophils, traditionally recognized for their role in parasitic infections and allergic inflammation, express TLR2 which enables them to recognize a broad spectrum of microbial components, particularly bacterial lipoproteins and lipopeptides. The heterodimerization of TLR2 with either TLR1 or TLR6 expands its ligand repertoire, allowing recognition of triacylated and diacylated lipopeptides, respectively, as well as other microbial molecules such as lipoteichoic acid and peptidoglycan. Studies using human eosinophilic EoL-1 cells have demonstrated that TLR2 activation induces pro-inflammatory responses and upregulates inflammasome components such as NLRC4 and NLRP3, which are involved in caspase-1 activation and IL-1β secretion, key mediators in inflammation (7). Furthermore, TLR2 expression on eosinophils is modulated during allergic airway disease, with NLRC4-deficient mice showing decreased eosinophil numbers and impaired Th2 responses, indicating that TLR2 and its downstream inflammasome pathways contribute to eosinophil function and immune regulation (7). TLR2 is also expressed in other innate immune cells such as neutrophils, macrophages, and dendritic cells, where it recognizes diverse PAMPs and triggers inflammatory cascades. For instance, neutrophils respond to TLR2 ligands by producing IL-1β via NLRP3 inflammasome activation, a process dependent on bacterial factors like Helicobacter pylori type IV secretion system and motility (38). In macrophages, TLR2 engagement leads to activation of signaling pathways including NF-κB and MAPKs, resulting in cytokine production and cellular activation (39). The recognition of PAMPs by TLR2 on eosinophils and other immune cells thus represents a crucial mechanism for sensing microbial presence and orchestrating appropriate immune responses (As shown in **Figure 2**).

The interaction between TLR2 signaling and IgE immune complexes (ICs) in eosinophils represents a sophisticated mechanism modulating eosinophilic responses during parasitic infections and allergic inflammation. IgE ICs, formed by antigen-specific IgE bound to its cognate antigen, engage Fcϵ receptors on eosinophils, notably FcϵRII (CD23), leading to cellular activation. Recent evidence indicates that ovalbumin (Ova)-IgE ICs can induce the expression of NLRC4 inflammasome components, caspase-1 activation, and IL-1β secretion in human eosinophilic EoL-1 cells via TLR2 signaling (40). This TLR2-dependent pathway also upregulates granule proteins such as MMP9, TIMP1, and TIMP2, which are involved in tissue remodeling and inflammation. While these observations are compelling, they derive from a single cell line (EoL-1) and may not fully recapitulate the responses of primary human eosinophils or murine eosinophils. The use of ovalbumin as a model antigen, while convenient, also raises questions about the generalizability of these findings to parasite-specific IgE responses, where antigen complexity and valency may differ substantially. Notably, the presence of Ova-IgE ICs suppresses the NLRC4 inflammasome activation induced by TLR2 ligands, suggesting a regulatory feedback mechanism. Moreover, co-stimulation with TLR2 ligands and Ova-IgE ICs enhances eosinophil degranulation, as evidenced by increased CD63 expression, a marker of granule exocytosis (40). This synergy between TLR2 and IgE ICs amplifies the release of inflammatory mediators, contributing to the pathophysiology of eosinophil-associated diseases such as asthma, chronic eosinophilic pneumonia, and parasitic infections. Beyond eosinophils, TLR2 activation synergizes with other receptors to modulate immune responses; for example, in dendritic cells, co-activation of TLR2 and macrophage-inducible C-type lectin receptor (Mincle) by bacterial components leads to enhanced IL-10 production (41). Similarly, TLR2 engagement in neutrophils and macrophages promotes the secretion of pro-inflammatory cytokines, including TNF-α and IL-6, often in cooperation with other PRRs and signaling pathways (42, 43). The spatial organization of TLR2 with other receptors such as FcγR and Dectin-1 on immune cell membranes further modulates the magnitude and quality of signaling crosstalk, influencing the balance between pro- and anti-inflammatory outcomes (44, 45). Collectively, these findings underscore the critical role of TLR2 in concert with IgE ICs and other receptors in orchestrating eosinophil activation, degranulation, and inflammatory mediator release, which are central to immune defense and immunopathology in parasitic and allergic diseases (As shown in **Figure 2**).

#### 2.2.3 Changes in the expression of degranulation marker CD63

CD63 is a well-recognized marker of eosinophil degranulation, reflecting the activation status and release of cytotoxic granule contents that contribute to inflammatory responses in tissues such as the lung. The interaction between IgE immune complexes (IgE ICs) and toll-like receptor 2 (TLR2) has been shown to synergistically enhance the proportion of CD63-positive eosinophils, indicating a heightened state of degranulation. Specifically, studies using the human eosinophilic cell line EoL-1 demonstrated that ovalbumin (Ova)-IgE ICs, when combined with TLR2 ligands, significantly increased the number of CD63+ cells compared to stimulation with native IgE alone. This suggests that IgE ICs can potentiate eosinophil activation through FcεRII and TLR2 co-signaling pathways, leading to amplified degranulation responses (6). The upregulation of CD63 is indicative of the exocytosis of eosinophilic granules containing matrix metalloproteinases (such as MMP9), eosinophil cationic protein (ECP), and tissue inhibitors of metalloproteinases (TIMPs), which play crucial roles in tissue remodeling and inflammation.

The degranulation process marked by CD63 expression is not merely a bystander event but exerts significant influence on pulmonary tissue inflammation. The release of granule proteins can directly damage lung parenchyma, increase vascular permeability, and recruit additional inflammatory cells, thereby exacerbating tissue injury and perpetuating chronic inflammation. In parasitic infections and allergic conditions such as eosinophilic pneumonia and asthma, this degranulation-driven inflammation contributes to disease pathology. Moreover, the regulatory role of IgE ICs in modulating NLRC4 inflammasome activation further adds complexity to the inflammatory milieu; while IgE ICs promote degranulation, they also appear to suppress NLRC4 inflammasome-mediated IL-1β secretion, suggesting a nuanced balance between pro-inflammatory and regulatory signals within eosinophils (6).

Therapeutic modulation of CD63 expression and eosinophil degranulation has been explored in the context of chronic inflammation. For instance, the novel Keap1 inhibitor ZINC-592 has demonstrated the ability to dose-dependently inhibit CD63 expression in IgE-FcϵRI-stimulated basophils, which share functional similarities with eosinophils in IgE-mediated responses. This inhibition correlates with reduced proinflammatory cytokine production and attenuation of chronic inflammatory states associated with viral infections. The underlying mechanism involves the Keap1/Nrf2 signaling pathway, which regulates oxidative stress responses and inflammation. By suppressing CD63 expression and consequent degranulation, agents like ZINC-592 may mitigate tissue damage and inflammation in IgE-driven diseases (46). Thus, targeting CD63 and the degranulation process represents a promising strategy to control eosinophil-mediated lung inflammation and improve outcomes in parasitic and allergic pulmonary disorders.

In summary, the expression changes of the degranulation marker CD63 in eosinophils are critically influenced by the interplay between IgE immune complexes and TLR2 signaling, which enhances eosinophil activation and degranulation. This process amplifies pulmonary inflammation and tissue remodeling, contributing to the pathogenesis of eosinophilic lung diseases. Concurrently, modulation of CD63 expression through pharmacologic agents targeting pathways such as Keap1/Nrf2 offers potential therapeutic avenues to regulate eosinophilic inflammation and protect lung tissue integrity. Understanding these mechanisms deepens insight into the immune regulation of eosinophil function in parasitic infections and allergic inflammation.

### 2.3 The role of NLRC4 inflammasome in eosinophils

#### 2.3.1 Structure and activation mechanism of the NLRC4 inflammasome

The NLRC4 inflammasome is a critical cytosolic multiprotein complex belonging to the NOD-like receptor (NLR) family, playing a pivotal role in innate immune defense against intracellular bacterial pathogens. Structurally, NLRC4 is characterized by a tripartite domain architecture comprising an N-terminal caspase activation and recruitment domain (CARD), a central nucleotide-binding oligomerization domain (NOD or NACHT), and a C-terminal leucine-rich repeat (LRR) domain. This modular structure enables NLRC4 to act as an adaptor and effector molecule, orchestrating inflammasome assembly and downstream signaling. Unlike other inflammasome sensors such as NLRP3, NLRC4 is constitutively expressed in resting macrophages and does not require inflammatory priming for its basal presence, although its expression can be upregulated by Toll-like receptor (TLR) signaling pathways, particularly through p38 MAP kinase activation in murine macrophages, which enhances its responsiveness to immunoevasive ligands (47, 48).

A unique feature of NLRC4 inflammasome activation is its reliance on upstream NAIP (NLR family apoptosis inhibitory proteins) receptors that serve as direct sensors for specific bacterial ligands. NAIPs recognize conserved components of bacterial type III secretion systems (T3SS), such as inner rod and needle proteins, and flagellin, a major structural protein of bacterial flagella (49, 50). Upon ligand binding, NAIPs undergo a conformational change from an inactive wide-open state to an active form capable of nucleating NLRC4 activation. Cryo-electron microscopy (cryo-EM) studies have elucidated that unliganded NAIP5 adopts a wide-open conformation with an exposed nucleating surface accessible for recruiting inactive NLRC4. Ligand engagement induces a rotation (~20°) of the winged helix domain (WHD) of NAIP5, which sterically clashes with inactive NLRC4, triggering NLRC4’s conformational transition to an active state. This conformational rearrangement stabilizes the NAIP5-NLRC4 complex through interactions involving the 17–18 loop of NAIP5, facilitating the nucleation and oligomerization of NLRC4 protomers into a wheel-like inflammasome structure (49, 51).

The assembly of the NLRC4 inflammasome is initiated by the binding of a single NAIP molecule to its cognate bacterial ligand, which then recruits and activates multiple NLRC4 molecules through ATPase-dependent oligomerization. This process is catalyzed by factors such as PELO, a ribosome-rescue factor that activates the ATPase activity of NLRC4, promoting sequential oligomerization and proper inflammasome assembly. PELO acts as a catalyzer rather than a structural component, highlighting a regulatory layer in NLRC4 inflammasome formation (52). Additionally, post-translational modifications, including deacetylation of NLRC4 by SIRT3, have been shown to promote inflammasome activation by enhancing NLRC4 oligomerization and ASC speck formation (53). Phosphorylation events mediated by kinases such as LRRK2 also modulate NLRC4 function, influencing the differential secretion of IL-1β and IL-18 cytokines during inflammasome activation (54).

The ligand specificity of NAIPs confers recognition of bacterial flagellin and T3SS components with high fidelity. For instance, flagellin recognition by NAIP5 involves a “trap and lock” mechanism, where the FliC-D0C domain of flagellin is first trapped by a hydrophobic pocket in NAIP5 and subsequently locked by the insertion domain and C-terminal tail, stabilizing the complex and facilitating the conformational changes necessary for NLRC4 activation (51). This precise molecular recognition ensures that the inflammasome is activated only upon detection of bona fide pathogenic signals.

Following activation, NLRC4 oligomerizes into a large wheel-like structure that recruits the adaptor protein ASC and pro-caspase-1, leading to caspase-1 activation. Activated caspase-1 processes pro-inflammatory cytokines IL-1β and IL-18 and cleaves gasdermin D to induce pyroptosis, a lytic form of programmed cell death that eliminates infected cells and alerts the immune system (55, 56). The NLRC4 inflammasome thus serves as a rapid-response platform for detecting intracellular bacterial components and initiating potent inflammatory responses.

In summary, the NLRC4 inflammasome is structurally defined by its CARD, NACHT, and LRR domains, with activation tightly regulated by ligand recognition through NAIPs, conformational changes, ATPase-driven oligomerization, and post-translational modifications. This sophisticated mechanism enables the host to detect bacterial virulence factors such as flagellin and T3SS proteins, assemble the inflammasome complex, and trigger downstream immune defenses to control infection and maintain homeostasis. Understanding these structural and mechanistic details provides crucial insights into the inflammasome’s role in host defense and its implications in infectious diseases, autoinflammatory conditions, and cancer (50, 57, 58).

#### 2.3.2 NLRC4 inflammasome-mediated IL-1β release

Upon activation, the NLRC4 inflammasome orchestrates a critical innate immune response by facilitating the cleavage of pro-inflammatory cytokines, notably interleukin-1β (IL-1β). This process is mediated through the activation of caspase-1, which proteolytically cleaves the inactive pro-IL-1β precursor into its mature, secreted form. The NLRC4 inflammasome is typically activated in response to cytosolic detection of bacterial components such as flagellin and type III secretion system proteins, with NAIP proteins serving as key sensors that engage NLRC4 to form the inflammasome complex. Upon assembly, the inflammasome recruits and activates caspase-1, which not only processes pro-IL-1β but also cleaves gasdermin D (GSDMD), leading to pyroptosis and the release of IL-1β through GSDMD pores (53, 59, 60). The regulation of NLRC4 inflammasome activation involves multiple layers, including post-translational modifications such as deacetylation by SIRT3, which promotes inflammasome assembly and function (53), and phosphorylation by kinases like LRRK2 that differentially modulate IL-1β and IL-18 secretion (54). Additionally, accessory proteins such as the E3 ubiquitin ligase HUWE1 mediate polyubiquitination of NLRC4, facilitating inflammasome assembly and sustained caspase-1 activity (61). The role of cathepsins and gasdermin D has also been elucidated as essential for optimal IL-1β secretion downstream of NLRC4 activation (59).

In the context of parasitic infections and pulmonary inflammation, IL-1β released via NLRC4 inflammasome activation exhibits a dual role. On one hand, IL-1β is pivotal in host defense by promoting inflammatory responses that facilitate pathogen clearance. For example, in Trypanosoma cruzi infection, NLRC4 activation leads to IL-1β and nitric oxide production, which are crucial for controlling parasite replication (62). Similarly, in fungal infections such as paracoccidioidomycosis, NLRC4 modulates IL-1β release, influencing disease susceptibility and immune effector mechanisms (63). On the other hand, excessive or dysregulated IL-1β secretion can exacerbate tissue inflammation and damage, contributing to the pathogenesis of inflammatory diseases. Elevated NLRC4 inflammasome activity and IL-1β levels have been implicated in chronic inflammatory conditions including adult-onset Still’s disease (64), diabetic nephropathy (65, 66), and neuroinflammation following intracerebral hemorrhage (67). Although these studies underscore the broad pathological relevance of NLRC4 inflammasome dysregulation, their direct applicability to eosinophil-mediated pulmonary inflammation during parasitic infection is limited by differences in cell types, inflammatory triggers, and tissue contexts. These findings are discussed here as potential parallels that warrant investigation in the pulmonary eosinophilic setting, rather than as direct evidence. In pulmonary fibrosis and other interstitial lung diseases, inflammasome-driven IL-1β release is linked to fibrotic progression, although NLRC4 activation levels may vary compared to other inflammasomes like NLRP3 and AIM2 (68). The pro-inflammatory cytokine IL-1β thus acts as a double-edged sword in parasitic eosinophilic lung diseases, where it contributes to both protective immunity and inflammatory pathology.

Mechanistically, NLRC4 inflammasome activation leads to caspase-1 cleavage, which processes pro-IL-1β to its active form, facilitating its secretion. This secretion is dependent on gasdermin D-mediated pore formation and is enhanced by cathepsin activity, which acts downstream of inflammasome assembly (59). Furthermore, signaling pathways such as p38 MAPK have been shown to regulate NLRC4 expression and caspase-1 activity, thereby modulating IL-1β production in macrophages exposed to inflammatory stimuli like ionizing radiation (69). The interplay between NLRC4 and other inflammasomes, such as NLRP3, can be complex; for instance, in certain infections, NLRP3 may compensate for suboptimal NLRC4 activation to maintain IL-1β secretion (60, 70). Additionally, immune complexes involving IgE can modulate NLRC4 inflammasome activation and IL-1β release in eosinophils, influencing allergic and parasitic responses in the lung.

In summary, the NLRC4 inflammasome mediates IL-1β release through caspase-1 activation and gasdermin D-dependent secretion, playing a critical role in the immune response to parasitic infections and lung inflammation. IL-1β functions dually as a mediator of host defense and a contributor to inflammatory pathology, underscoring the importance of tightly regulated NLRC4 inflammasome activity in maintaining pulmonary immune homeostasis and preventing excessive tissue damage.

#### 2.3.3 Proposed regulatory effects of IgE immune complexes on NLRC4 inflammasome: evidence from cellular models

This section provides the primary and most detailed description of the experimental evidence supporting the proposed IgE IC-TLR2-NLRC4 pathway. Subsequent sections will build upon this mechanistic foundation rather than restating it.

IgE immune complexes (ICs) have been reported to exert a significant inhibitory effect on the activation of the NLRC4 inflammasome in EoL-1 cells, thereby potentially modulating the intensity of inflammatory responses. The NLRC4 inflammasome, a cytosolic multiprotein complex primarily recognized for its role in detecting bacterial flagellin and initiating caspase-1-dependent maturation of pro-inflammatory cytokines such as interleukin-1β (IL-1β), plays a crucial role in innate immune responses and inflammation. In eosinophils, which are key effector cells in parasitic infections and allergic inflammation, NLRC4 activation leads to the release of IL-1β and other inflammatory mediators that contribute to tissue damage and disease pathology. Recent research utilizing a human eosinophilic cell line (EoL-1) demonstrated that ovalbumin-specific IgE immune complexes (Ova-IgE ICs) can induce the expression of NLRC4 inflammasome components, including NLRC4 itself, caspase-1, and intracellular IL-1β, through Toll-like receptor 2 (TLR2) signaling pathways. However, paradoxically, when the NLRC4 inflammasome is activated in the presence of these IgE ICs, the typical pro-inflammatory responses, including IL-1β secretion and release of granule proteins such as matrix metalloproteinase 9 (MMP9), tissue inhibitors of metalloproteinases (TIMP1 and TIMP2), are reportedly suppressed. This raises the possibility that IgE ICs may fine-tune the inflammatory output by dampening excessive inflammasome activation, thus potentially preventing overwhelming inflammation (6).However, it is critical to note that these findings are currently limited to a single *in vitro* system. If confirmed in primary cells and *in vivo*, this regulatory mechanism would be of substantial immunological importance. At present, this hypothesis awaits rigorous testing in physiologically relevant models.

The regulatory mechanism mediated by IgE ICs is of substantial immunological importance, particularly in maintaining immune homeostasis and protecting tissues from excessive inflammatory damage. By modulating NLRC4 inflammasome activity, IgE ICs help balance the need for effective immune defense against pathogens and parasites with the necessity to limit collateral tissue injury caused by hyperinflammation. This balance is especially critical in eosinophil-associated diseases such as chronic eosinophilic pneumonia, eosinophilic esophagitis, eosinophilic granulomatosis, parasitic infections, allergy, and asthma, where dysregulated eosinophil activation and inflammasome signaling contribute to pathology. The ability of IgE ICs to suppress NLRC4 inflammasome-driven IL-1β production and granule protein release may reduce tissue inflammation and fibrosis, thereby mitigating disease severity. Moreover, the IgE ICs’ engagement of FcεRII (CD23) receptors on eosinophils initiates signaling cascades that further regulate gene expression of inflammatory mediators, including MMP9 and TIMP2, which are involved in extracellular matrix remodeling and tissue repair processes. This dual role of IgE ICs in both activating and restraining eosinophilic responses underscores their potential as modulators of immune balance in parasitic and allergic lung diseases (6).

Collectively, these findings highlight a novel immunoregulatory pathway whereby IgE immune complexes modulate NLRC4 inflammasome activation and eosinophil function. The suppression of NLRC4 inflammasome activity by IgE ICs represents a critical checkpoint in controlling the magnitude and quality of inflammatory responses in the lung. Understanding this regulatory axis could provide new therapeutic insights for managing eosinophil-driven pulmonary disorders, where excessive inflammasome activation and IgE-mediated hypersensitivity coexist. Targeting the IgE IC-NLRC4 inflammasome interaction may offer strategies to restore immune equilibrium, reduce chronic inflammation, and prevent irreversible tissue damage in parasitic eosinophilic lung diseases and allergic conditions (6).

#### 2.3.4 Current evidence gaps and unresolved questions

Despite the intriguing observations from cellular models, several critical gaps limit the translation of these findings to parasitic eosinophilic lung disease:

First, the mechanistic evidence for the IgE IC-TLR2-NLRC4 axis is derived exclusively from the EoL-1 human eosinophilic cell line stimulated with Ova-IgE complexes. Primary human eosinophils and murine eosinophils may exhibit different signaling responses due to variations in receptor expression and downstream signaling components. Second, no *in vivo* studies have directly tested whether IgE ICs modulate NLRC4 inflammasome activity during helminth infection. Genetic deletion of FcϵRII, TLR2, or NLRC4 in murine models of parasitic pulmonary disease would be essential to establish causality. Third, the presence and functional significance of this pathway in human parasitic eosinophilic lung disease remain completely unexplored. Clinical studies correlating IgE IC levels with NLRC4 inflammasome activity and disease severity are urgently needed. Fourth, the relative contribution of this pathway compared to other well-established inflammasome pathways (particularly NLRP3) and type 2 immune mediators (IL-33, TSLP, alarmins) has not been defined. Addressing these gaps will require integrated approaches combining animal models, primary cell systems, and human clinical samples.

#### 2.3.5 NLRC4 in context: comparison with other inflammasome pathways in eosinophilic pulmonary inflammation

The NLRC4 inflammasome is one of several inflammasome complexes implicated in pulmonary inflammation. To provide appropriate context, it is essential to compare the evidence supporting NLRC4 with that for the more extensively studied NLRP3 inflammasome.

NLRP3 is the most well-characterized inflammasome in the lung, with established roles in allergic asthma, chronic obstructive pulmonary disease, and pulmonary fibrosis. NLRP3 activation in response to diverse stimuli (ATP, crystalline substances, environmental pollutants) triggers IL-1β and IL-18 release, promoting type 2 inflammation and airway remodeling. In parasitic infections, NLRP3 has been implicated in the host response to various helminths. The evidence supporting NLRP3 in eosinophilic pulmonary disease is considerably more extensive than that for NLRC4, with multiple *in vivo* studies demonstrating its pathological or protective roles.

In contrast, NLRC4 is primarily recognized for its role in detecting bacterial flagellin and type III secretion system components. Its relevance to eosinophil biology is a more recent discovery, with current evidence primarily derived from *in vitro* studies. The rationale for proposing a role for NLRC4 in parasitic eosinophilic lung disease is based on the following considerations: (1) parasites may translocate bacterial components or alter the lung microbiome, providing NLRC4 ligands; (2) eosinophils constitutively express NLRC4, enabling rapid responses; and (3) preliminary data suggest a functional link between IgE ICs, TLR2, and NLRC4. However, these remain hypotheses requiring experimental verification.

Other inflammasomes including AIM2 and NLRP1 have also been studied in pulmonary inflammation, though their roles in eosinophilic disease are less defined.

In summary, while this review focuses on NLRC4 due to its emerging connection to IgE-mediated eosinophil regulation, NLRP3 remains the most consistently implicated inflammasome in pulmonary type 2 inflammation. The NLRC4-centric framework presented here is intended as a hypothesis-generating perspective, not as a claim of dominance over other inflammasome pathways.

### 2.4 Comprehensive regulatory network of immune complexes and NLRC4 inflammasome interaction

#### 2.4.1 Cross-regulation between immune complexes and PRR signaling pathways

The mechanistic details of IgE IC-mediated NLRC4 inflammasome modulation, including the roles of FcϵRII, TLR2, and downstream signaling cascades, have been described in detail in Section 2.3.3. The present section focuses on integrating this pathway with the broader context of PRR signaling networks and eosinophil biology.

Immunoglobulin E (IgE) immune complexes (ICs) exert a significant modulatory effect on innate immune responses, particularly through their interactions with pattern recognition receptors (PRRs) such as Toll-like receptors (TLRs) and nucleotide-binding oligomerization domain-like receptors (NLRs). In the context of eosinophilic immune responses, IgE ICs engage with Fc receptors, notably FcϵRII (CD23), and TLR2 to orchestrate downstream signaling cascades that influence the activity of the NLRC4 inflammasome, a cytosolic PRR complex critical for innate immune defense.

As described in Section 2.3.3, experimental evidence using EoL-1 cells has demonstrated that Ova-IgE ICs, through FcϵRII and TLR2 co-signaling, can induce NLRC4 inflammasome components while also exerting a suppressive effect on fully activated inflammasomes. This cross-talk modulates eosinophil degranulation and tissue remodeling functions via regulation of MMP9, TIMPs, and ECP expression. These findings underscore the integrated signaling network where IgE ICs, through FcϵRII and TLR2, converge on the regulation of NLRC4 inflammasome activity and eosinophil effector functions.

Co-stimulation with TLR2 ligands and IgE ICs further enhances eosinophil degranulation, as indicated by increased CD63 expression, a marker of granule exocytosis. These findings underscore the integrated signaling network where IgE ICs, through FcϵRII and TLR2, converge on the regulation of NLRC4 inflammasome activity and eosinophil effector functions, thereby fine-tuning the immune response in parasitic infections, allergic inflammation, and eosinophil-associated diseases (71).

At the molecular level, this cross-regulation exemplifies how immune complexes can influence PRR signaling pathways to achieve a balanced immune response. The FcϵRII receptor, traditionally associated with IgE-mediated allergic responses, acts as a modulator of TLR2-driven inflammasome activation, suggesting a novel axis of immune regulation. This interaction may involve shared adaptor proteins and signaling intermediates that integrate signals from Fc receptors and TLRs, ultimately affecting the assembly and activation of the NLRC4 inflammasome complex. Such integration is critical because PRRs like TLR2 initiate pro-inflammatory signaling upon recognition of pathogen-associated molecular patterns (PAMPs), while inflammasomes like NLRC4 amplify inflammatory responses through caspase-1-dependent cytokine maturation. The ability of IgE ICs to modulate these pathways highlights the complexity of immune regulation, where antibody-mediated recognition interfaces with innate immune sensing to calibrate the magnitude and quality of the inflammatory response.

Furthermore, the comprehensive regulation of eosinophil function by immune complexes and PRR signaling extends beyond cytokine production to include the modulation of granule protein release and matrix remodeling enzymes. The upregulation of MMP9 and TIMPs suggests that IgE ICs influence tissue remodeling processes, which are crucial in chronic inflammatory conditions such as asthma and eosinophilic pneumonia. The interplay between FcϵRII and TLR2 signaling pathways may also affect eosinophil survival, chemotaxis, and interaction with other immune cells, thereby shaping the overall immune milieu.

At the molecular level, this cross-regulation exemplifies how immune complexes can influence PRR signaling pathways to achieve a balanced immune response. The FcϵRII receptor, traditionally associated with IgE-mediated allergic responses, appears to act as a modulator of TLR2-driven inflammasome activation. This interaction may involve shared adaptor proteins and signaling intermediates that integrate signals from Fc receptors and TLRs, ultimately affecting the assembly and activation of the NLRC4 inflammasome complex. Such integration is critical because PRRs like TLR2 initiate pro-inflammatory signaling upon recognition of pathogen-associated molecular patterns (PAMPs), while inflammasomes like NLRC4 amplify inflammatory responses through caspase-1-dependent cytokine maturation. This integrative model, while speculative, suggests potential mechanisms by which IgE ICs may influence eosinophil function beyond the core pathway described in Section 2.3.3.

In summary, IgE immune complexes engage FcϵRII and TLR2 to orchestrate a finely tuned regulatory network that modulates NLRC4 inflammasome activity and eosinophil effector functions. This cross-talk represents a critical mechanism by which adaptive immune components (IgE ICs) influence innate immune receptors (PRRs) to maintain immune homeostasis and prevent excessive inflammation. Understanding these interactions provides valuable insights into the pathogenesis of eosinophil-associated diseases and may inform the development of targeted immunomodulatory therapies (71).

#### 2.4.2 Insights from other inflammatory conditions: a comparative perspective

The core mechanistic pathway—IgE IC engagement of FcϵRII, co-stimulation with TLR2, and modulation of NLRC4 inflammasome activity—has been described in detail in Section 2.3.3. Rather than restating this mechanism, the present section focuses on critically evaluating the translatability of findings from other inflammatory conditions to parasitic eosinophilic lung disease. It is important to emphasize that the pathway described in Section 2.3.3 is derived primarily from a single *in vitro* study using EoL-1 cells. Extrapolation of this pathway to other disease contexts must be undertaken with appropriate caution.

While this review focuses on parasitic eosinophilic lung disease, relevant mechanistic insights have also emerged from studies of other inflammatory conditions. It is important to note, however, that findings from these systems should be extrapolated with caution, as the inflammatory triggers, chronicity, and tissue microenvironments differ substantially from parasitic infections.

IgE immune complexes (ICs) play a paradoxical role in modulating inflammation, exhibiting both pro-inflammatory and anti-inflammatory effects depending on context. Studies in allergic asthma have demonstrated IgE-mediated pathways that may offer parallel insights (72). However, the nature of the antigen (environmental allergen versus parasitic antigen), duration of exposure (intermittent versus chronic), and co-existence of live parasites create distinct immunological contexts. The mechanistic details of IgE IC signaling—involving FcϵRII, TLR2, and NLRC4—have been presented in Section 2.3.3 and are not repeated here.

As detailed in Section 2.3.3, Ova-IgE ICs have been shown to both induce and suppress NLRC4 inflammasome responses in EoL-1 cells, suggesting a complex feedback mechanism (73). The interplay between IgE ICs and inflammasome pathways thus provides a delicate balance between promoting necessary immune defense and preventing tissue damage from overactive inflammation. Investigations in cancer have revealed inflammasome-mediated regulation of tumor immunity; however, the role of NLRC4 in antitumor responses is fundamentally different from its potential function in antiparasitic defense, where tissue-invasive larvae and ongoing pathogen replication present unique challenges. Similarly, reports linking NLRC4 inflammasome to diabetic nephropathy, intracerebral hemorrhage, and adult-onset Still’s diseasehave provided valuable knowledge about NLRC4 biology and its regulation by post-translational modifications. Nevertheless, the relevance of these findings to eosinophil biology in the lung remains unclear, as these conditions involve different cell types (renal tubular cells, neurons, monocytes) and distinct inflammatory triggers.

In summary, while comparative discussion enriches our understanding of NLRC4 inflammasome biology, the direct translatability of findings from non-parasitic or non-eosinophilic systems to parasitic eosinophilic lung disease is limited. Throughout this review, we have attempted to distinguish between disease-relevant evidence and broader mechanistic knowledge, acknowledging that the latter serves primarily as a foundation for generating hypotheses rather than establishing disease-specific conclusions.

The regulatory capacity of immune complexes extends to the modulation of inflammasome activity, particularly NLRC4, offering new perspectives on maintaining immune homeostasis. Immune complexes can influence inflammasome activation, thereby fine-tuning the inflammatory milieu. For example, the suppression of NLRC4 inflammasome activation by IgE ICs may prevent excessive IL-1β release, which is critical in controlling the intensity and duration of inflammation (73). This modulatory effect aligns with the broader concept of balanced immune responses, where pro-inflammatory signals such as tumor necrosis factor-alpha (TNF-α), interleukin-6 (IL-6), and IL-1 are counterbalanced by anti-inflammatory mediators like IL-10 and transforming growth factor-beta (TGF-β) to avoid chronic inflammation and tissue injury (74). Moreover, the involvement of immune complexes in controlling inflammasome activity may have implications beyond allergic diseases, potentially influencing chronic inflammatory conditions and autoimmunity where dysregulated inflammasome activation contributes to pathogenesis.

Further insights into the immune complex-mediated regulation of inflammation highlight the importance of receptor interactions and downstream signaling pathways. Fc gamma receptors (FcγRs), which bind IgG-containing immune complexes, have been implicated in modulating inflammatory responses in vascular injury and atherosclerosis models, demonstrating that activating FcγRIII can both promote and limit inflammation depending on disease stage (75). This dichotomy underscores the complexity of immune complex signaling in balancing inflammation. Additionally, anti-inflammatory cytokines such as IL-10 play a crucial role in limiting immune complex-induced tissue damage by suppressing neutrophil infiltration and pro-inflammatory cytokine expression, as evidenced in murine models of type III vasculitis (76). These findings suggest that immune complexes, through their interactions with various Fc receptors and cytokine networks, orchestrate a finely tuned inflammatory response that can either resolve inflammation or contribute to pathology if dysregulated.

The therapeutic potential of targeting immune complex-mediated pathways is increasingly recognized. For example, modulation of IgE responses through anti-IgE therapies or induction of blocking IgG antibodies can prevent allergen-induced inflammation by interfering with IgE-allergen interactions (72). Moreover, novel small molecules such as ZINC-592 have shown promise in mitigating IgE-mediated chronic inflammation by modulating the Keap1/Nrf2 signaling axis, highlighting the potential for pharmacological intervention in IgE-related immune complex inflammation (46). Understanding the dual roles of IgE ICs and their regulation of inflammasome activity thus opens avenues for developing therapies that restore immune balance by enhancing anti-inflammatory mechanisms while preserving host defense.

In summary, IgE immune complexes exhibit a dualistic role in inflammation, capable of both promoting and suppressing immune responses through mechanisms involving Fc receptor engagement and inflammasome modulation. The regulation of NLRC4 inflammasome activity by IgE ICs represents a critical checkpoint in maintaining immune homeostasis, preventing excessive inflammation and tissue damage. This balance between pro- and anti-inflammatory signals mediated by immune complexes provides a conceptual framework for understanding and potentially manipulating immune responses in parasitic infections, allergic diseases, and other inflammatory conditions. Further research into these pathways may yield novel therapeutic strategies aimed at harnessing the immunomodulatory functions of IgE immune complexes and inflammasome regulation (46, 72–76).

#### 2.4.3 Regulation of expression of related proteases and inhibitory factors

Matrix metalloproteinase 9 (MMP9) and its endogenous inhibitors, tissue inhibitors of metalloproteinases 1 and 2 (TIMP1 and TIMP2), play pivotal roles in tissue remodeling and inflammation, particularly within the pulmonary microenvironment. MMP9, a member of the gelatinase subgroup of MMPs, is capable of degrading components of the extracellular matrix (ECM), such as type IV collagen, thereby facilitating structural remodeling during inflammatory responses and repair processes. However, excessive or dysregulated MMP9 activity can contribute to pathological tissue damage and fibrosis. TIMP1 and TIMP2 serve as critical regulators by binding to active MMP9 and other MMPs, thereby inhibiting their proteolytic activity and maintaining ECM homeostasis. The balance between MMP9 and TIMPs is thus essential for controlled tissue remodeling and resolution of inflammation in the lung. Dysregulation of this balance has been implicated in various pulmonary diseases characterized by inflammation and fibrosis, including parasitic eosinophilic lung diseases. Immune complexes (ICs) involving IgE (IgE ICs), which form during parasitic infections, have been shown to modulate the expression of these proteases and their inhibitors. IgE ICs can engage Fc epsilon receptors on immune cells, triggering signaling cascades that influence gene expression profiles related to protease production. This modulation affects the local microenvironment by altering ECM turnover, thereby impacting both tissue repair and injury. Specifically, IgE ICs may upregulate MMP9 expression, promoting ECM degradation necessary for immune cell infiltration and clearance of parasitic elements, while simultaneously influencing TIMP1 and TIMP2 levels to prevent excessive tissue destruction. Such regulatory effects on protease expression by IgE ICs are critical in balancing effective immune defense with preservation of lung tissue integrity. Understanding these mechanisms provides insights into how parasitic infections orchestrate immune responses that involve proteolytic remodeling and highlights potential therapeutic targets to mitigate lung damage while enhancing repair processes in eosinophilic pulmonary diseases (77, 78).

### 2.5 Clinical significance and research prospects of immune regulation mechanisms in parasitic eosinophilic pneumonia

#### 2.5.1 Immunoregulatory mechanisms and their implications for disease diagnosis and treatment

Understanding the immunoregulatory mechanisms involving IgE immune complexes (ICs) and the NLRC4 inflammasome offers promising avenues for developing targeted immunomodulatory strategies in parasitic eosinophilic lung diseases. IgE ICs, formed by the binding of antigen-specific IgE to allergens or pathogens, have been shown to modulate eosinophilic responses through interaction with innate immune receptors such as FcϵRII and Toll-like receptor 2 (TLR2). Recent studies demonstrate that ovalbumin (Ova)-IgE ICs induce the expression of NLRC4 inflammasome components, including NLRC4 itself, caspase-1, and interleukin-1β (IL-1β), as well as granule proteins like MMP9, TIMP1, and TIMP2 via TLR2 signaling in eosinophils. Interestingly, activation of the NLRC4 inflammasome in the presence of these ICs suppresses some of these inflammatory responses, indicating a complex regulatory feedback that balances inflammation and tissue remodeling. This dual role suggests that manipulating IgE ICs or NLRC4 inflammasome pathways could fine-tune eosinophilic inflammation, providing a targeted approach to diseases such as chronic eosinophilic pneumonia, eosinophilic esophagitis, and parasitic infections (6).

From a diagnostic perspective, the expression levels of inflammasome components and granule proteins induced by IgE ICs may serve as potential biomarkers to monitor disease activity and response to therapy. The co-stimulation of TLR2 ligands with IgE ICs enhances eosinophil degranulation, as evidenced by increased CD63+ cells, a marker of degranulation, suggesting that measuring these markers could reflect the inflammatory status in eosinophilic lung diseases. Furthermore, the involvement of FcϵRII in mediating these effects highlights the receptor as a potential therapeutic target to modulate eosinophil activation without broadly suppressing the immune system (6).

Beyond the direct effects of IgE ICs and NLRC4 inflammasome, broader principles of immune regulation, including the maintenance of immune tolerance via regulatory T cells (79), may inform therapeutic strategies to mitigate eosinophilic inflammation. However, application of these general principles to parasitic eosinophilic lung disease requires disease-specific investigation. Similarly, advances in biomarker discovery, including the identification of lncRNA signatures associated with inflammation (80, 81), may eventually contribute to precision medicine approaches, though such applications remain speculative in the context of parasitic eosinophilic lung disease.

Therapeutically, targeting the IgE IC-NLRC4 axis offers a novel immunomodulatory strategy. Modulation of FcϵRII signaling or TLR2 pathways could attenuate pathological eosinophil activation while preserving host defense. Additionally, leveraging the regulatory functions of inflammasomes may help balance pro- and anti-inflammatory responses. Cell-based therapies that enhance regulatory immune cell populations or manipulate macrophage polarization have shown promise in related inflammatory lung diseases, suggesting potential applicability in eosinophilic parasitic infections (82). Moreover, the use of biologics targeting IgE or inflammasome components could be explored to achieve selective immune modulation.

In summary, elucidating the mechanisms by which IgE ICs interact with the NLRC4 inflammasome enriches our understanding of eosinophilic immune regulation and provides a framework for developing targeted diagnostic and therapeutic strategies. The identification of specific biomarkers linked to these pathways could improve disease monitoring and guide personalized treatment. Future research integrating molecular, cellular, and clinical data will be pivotal in translating these insights into effective interventions for parasitic eosinophilic lung diseases.

#### 2.5.2 Limitations of existing treatments and directions for improvement

##### 2.5.2.1 Traditional antiparasitic and anti-inflammatory therapies: limitations potential of innovative therapies based on immune complex regulation mechanisms

Current therapeutic approaches for parasitic eosinophilic lung diseases primarily rely on antiparasitic agents and anti-inflammatory treatments, such as corticosteroids. While these strategies can be effective in many cases, several limitations hinder their overall efficacy and safety. Antiparasitic drugs, including praziquantel and albendazole, target the elimination of the causative helminths, as demonstrated in pulmonary paragonimiasis and visceral larva migrans caused by Ascaris suum, respectively (83, 84). However, these drugs may not fully resolve the inflammatory sequelae, and their efficacy depends on accurate and timely diagnosis, which is often challenging due to nonspecific clinical manifestations and frequent misdiagnoses as bacterial pneumonia or tuberculosis (85, 86). Additionally, prolonged inflammation mediated by eosinophils and immune complexes can cause tissue damage, which antiparasitic therapy alone does not address.

Anti-inflammatory treatments, predominantly corticosteroids, are used to control eosinophilic inflammation and prevent lung tissue damage, as seen in chronic eosinophilic pneumonia (87). However, steroid therapy carries risks of systemic side effects and may lead to immunosuppression, increasing susceptibility to secondary infections. Moreover, corticosteroids do not specifically target the underlying immune dysregulation involving IgE immune complexes and inflammasome activation, which are increasingly recognized as key drivers of pathology in parasitic eosinophilic lung diseases. For instance, the NLRC4 inflammasome has been implicated in mediating inflammatory responses during parasitic infections and related lung injury (88). The lack of targeted immunomodulatory therapies results in incomplete resolution of inflammation and potential relapses.

Furthermore, current treatments do not adequately address the heterogeneity of immune responses among patients, including variations in IgE levels, eosinophil activation, and inflammasome activity. This variability complicates the management of parasitic eosinophilic lung diseases and necessitates personalized therapeutic approaches. Diagnostic challenges, such as distinguishing parasitic infections from allergic or autoimmune eosinophilic lung disorders, further complicate treatment decisions (89, 90). Therefore, there is a critical need for therapies that can precisely modulate the immune pathways involved, minimize side effects, and improve patient outcomes.

##### 2.5.2.2 Potential of innovative therapies based on immune complex regulation mechanisms

The mechanistic basis for these therapeutic approaches—namely, the proposed IgE IC-FcϵRII-TLR2-NLRC4 signaling axis—has been described in detail in Section 2.3.3. The present section focuses on the translational and therapeutic implications of this pathway, rather than re-describing the underlying mechanism. Importantly, all therapeutic strategies discussed in this section are theoretical and based on the hypothetical pathway described in Section 2.3.3. *In vivo* validation is urgently needed before these approaches can be considered for clinical translation.

As discussed in Section 2.3.3, emerging evidence suggests that IgE immune complexes may modulate NLRC4 inflammasome activity in eosinophils, potentially influencing type 2 inflammation and tissue damage (88, 91). These observations, if validated *in vivo*, open avenues for novel therapeutic strategies that could specifically target immune complex-mediated pathways and inflammasome signaling.

One promising direction involves the development of biologics that neutralize IgE or disrupt IgE receptor interactions. Advances in understanding the structural dynamics of IgE and its receptors have led to the creation of next-generation anti-IgE molecules capable of disassembling preformed IgE: FcϵRI complexes, thereby rapidly attenuating allergic and parasitic inflammation (91). Such disruptive IgE inhibitors could potentially reduce eosinophil activation and downstream inflammatory cascades more efficiently than conventional therapies.

Targeting inflammasome components represents another innovative approach. The NLRC4 inflammasome, activated during parasitic infections and implicated in lung inflammation, can be modulated by pharmacological agents such as rottlerin, which inhibits PKCδ-mediated NLRC4 phosphorylation and subsequent inflammasome assembly, thereby reducing pyroptosis and inflammatory cytokine release (88). This mechanism suggests that selective inhibition of NLRC4 inflammasome activation could mitigate lung tissue injury without broadly suppressing immune function.

Moreover, strategies enhancing autophagy to promote degradation of inflammasome components, such as p62-dependent NLRC4 degradation, have shown protective effects against lung pyroptosis in experimental models (92) Modulating autophagy pathways may thus offer a complementary means to control inflammasome-driven inflammation.

The integration of immunoglobulin genetics and population diversity studies further supports personalized medicine approaches. Understanding individual variations in immunoglobulin alleles that influence antibody binding and immune responses can inform the design of tailored therapies and vaccines for parasitic infections (93). Additionally, leveraging computational methods and molecular dynamics simulations accelerates the discovery of selective inflammasome inhibitors, enhancing the precision of therapeutic interventions (94).

In summary, innovative therapies that regulate IgE immune complexes and modulate NLRC4 inflammasome activation hold significant potential to overcome the limitations of traditional antiparasitic and anti-inflammatory treatments. These approaches promise more targeted, effective, and safer management of parasitic eosinophilic lung diseases, ultimately improving patient outcomes and reducing disease burden.

#### 2.5.3 Future research priorities and technological development trends

##### 2.5.3.1 Further elucidation of the effects of different immune complex types on eosinophils

The research priorities outlined below are predicated on the hypothesis that the pathway described in Section 2.3.3 is physiologically relevant—a hypothesis that requires rigorous experimental testing. Understanding the differential impact of various immune complex (IC) types on eosinophil biology remains a critical frontier in parasitic eosinophilic lung diseases. As described in Section 2.3.3, recent studies using the EoL-1 cell line have demonstrated that Ova-IgE ICs can induce NLRC4 inflammasome components and modulate eosinophil degranulation markers through FcϵRII and TLR2 signaling pathways (6). This suggests that the nature of the antigen-specific IgE ICs may skew eosinophilic immune responses toward either pro-inflammatory or regulatory outcomes. However, the extent to which different IC isotypes (e.g., IgG, IgA, IgE) and their antigenic specificities influence eosinophil activation *in vivo* remains incompletely defined.

Moreover, the interplay between ICs and eosinophil-expressed inflammasomes, particularly NLRC4, requires further dissection to clarify how these complexes modulate inflammasome assembly and downstream signaling cascades. Given the heterogeneity of eosinophilic lung diseases, including parasitic infections, allergic bronchopulmonary aspergillosis, and idiopathic chronic eosinophilic pneumonia, delineating the specific contributions of diverse IC types to eosinophil function could reveal novel immunoregulatory checkpoints and therapeutic targets.

##### 2.5.3.2 Application of single-cell sequencing and high-throughput screening technologies for in-depth analysis of immune regulatory networks

Advanced single-cell RNA sequencing (scRNA-seq) and high-throughput screening technologies offer unprecedented resolution to unravel the complexity of immune regulatory networks governing eosinophilic inflammation in parasitic lung diseases. These technologies enable the characterization of cellular heterogeneity, identification of rare cell subsets, and mapping of dynamic transcriptional states within the pulmonary microenvironment. For example, scRNA-seq can dissect eosinophil subpopulations and their activation status, revealing differential expression of inflammasome components such as NLRC4, caspase-1, and cytokines like IL-1β in response to parasitic antigens or immune complexes (6, 7). High-throughput screening platforms, including CRISPR-Cas9-based genetic screens or small-molecule libraries, can identify novel regulators of eosinophil inflammasome activation and immune complex signaling pathways. Integration of these datasets with proteomics and epigenomics will further elucidate post-translational modifications (e.g., NLRC4 deacetylation by SIRT3) and transcriptional regulators (e.g., vitamin D receptor or TLR signaling) that fine-tune inflammasome assembly and function (53). Applying these cutting-edge methodologies to both animal models and human clinical samples will provide comprehensive insights into the immune networks orchestrating eosinophilic lung pathology and facilitate the discovery of biomarkers and precision therapies.

##### 2.5.3.3 Validation of mechanisms and therapeutic efficacy through combined animal models and clinical samples

Robust validation of mechanistic findings and therapeutic interventions necessitates the integration of well-characterized animal models with clinical specimen analyses. Animal models, such as murine infections with parasitic helminths (e.g., Trichinella spiralis) or bacterial pathogens (e.g., Salmonella typhimurium), have been instrumental in delineating the role of eosinophils and NLRC4 inflammasome activation in pulmonary inflammation and host defense (95, 96). These models allow controlled manipulation of immune components, including genetic deletion of NLRC4 or modulation of signaling pathways like TLR priming and SIRT3-mediated deacetylation, to assess their contributions to disease phenotypes (48, 53). Parallel analyses of clinical samples from patients with eosinophilic lung diseases, parasitic infections, or eosinophilic granulomatosis with polyangiitis provide translational relevance by confirming inflammasome activation markers, cytokine profiles, and immune complex presence *in vivo* (2, 97). Combining these approaches enables the evaluation of novel therapeutic strategies targeting IgE immune complexes, NLRC4 inflammasome components, or upstream regulators such as vitamin D receptor or LRRK2 kinase in mitigating eosinophilic inflammation. Furthermore, longitudinal studies incorporating bronchoalveolar lavage, blood eosinophil counts, and imaging can monitor treatment efficacy and disease progression. This integrative strategy will accelerate the translation of mechanistic insights into clinical interventions for parasitic eosinophilic lung diseases.

## 3 Conclusion

In conclusion, the intricate immunoregulatory mechanisms underlying parasitic eosinophilic pneumonia represent a compelling frontier in pulmonary immunology. Our analysis suggests that IgE immune complexes (ICs) may serve as pivotal modulators within this complex cellular signaling network. From an expert perspective, we propose that IgE ICs could orchestrate eosinophilic inflammation through engagement of FcϵRII and TLR2 pathways, potentially fine-tuning eosinophil activation and degranulation. A key hypothesis emerging from this review is that these immune complexes might exert a suppressive effect on the overactivation of the NLRC4 inflammasome, thereby potentially maintaining immune homeostasis and preventing excessive tissue damage. However, this framework remains speculative and requires rigorous experimental validation.

The NLRC4 inflammasome, through its mediation of IL-1β release, stands out as a potential central node in the inflammatory cascade characteristic of parasitic eosinophilic lung disease. The dynamic interplay between IgE ICs and the NLRC4 inflammasome may elucidate a previously underappreciated regulatory axis that integrates innate and adaptive immune signals to modulate disease severity and progression. It is important to recognize the hierarchy of evidence presented in this review. The strongest support for eosinophil effector functions and Th2/IgE responses comes from established *in vivo* models and human studies. The proposed IgE IC-TLR2-NLRC4 axis, in contrast, rests on preliminary *in vitro* observations that require validation in physiologically relevant systems. Readers should interpret the mechanistic model presented here as a hypothesis-generating framework rather than an established paradigm.

This nuanced understanding reconciles seemingly divergent research findings by positioning immune complexes not merely as pathological agents but as critical immunomodulatory entities that balance pro-inflammatory and anti-inflammatory forces within the pulmonary microenvironment.

Clinically, these insights suggest promising avenues for targeted interventions. Therapeutic strategies that might harness or mimic the regulatory functions of IgE ICs could potentially attenuate eosinophilic inflammation without compromising host defense, though such approaches remain theoretical at present. Moreover, modulating NLRC4 inflammasome activity offers a strategic target to curb IL-1β-driven pathology, which is often refractory to conventional treatments. However, the complexity of these signaling networks necessitates a cautious approach to avoid unintended immunosuppression or exacerbation of parasitic burden.

Looking forward, future research must delve deeper into the molecular intricacies governing IgE IC and NLRC4 inflammasome interactions. Advanced techniques such as single-cell transcriptomics and high-resolution imaging could unravel cell-specific signaling events and temporal dynamics that define disease phenotypes. Additionally, exploring genetic and environmental factors that influence this immunoregulatory axis will be critical to personalize therapeutic approaches. Integrating these mechanistic insights with clinical data will facilitate the development of novel immunomodulatory therapies that are both effective and safe.

In summary, the evolving understanding of immune complex-mediated regulation in parasitic eosinophilic pneumonia not only enriches our comprehension of disease pathogenesis but also lays a robust foundation for innovative treatment paradigms. Balancing the diverse research perspectives and findings, it is evident that targeting the IgE IC–NLRC4 inflammasome pathway holds significant promise. Continued interdisciplinary efforts will be essential to translate these scientific advances into tangible clinical benefits, ultimately improving patient outcomes in this challenging pulmonary disorder.
